# Intraoperative hemodynamic collapse revealing undiagnosed pheochromocytoma in a patient with neurofibromatosis type 1 and severe cervical deformity: a case report on perioperative management

**DOI:** 10.1186/s40981-026-00860-w

**Published:** 2026-05-11

**Authors:** Riko Ideyama, Chikashi Takeda, Yohei Chiwata, Yui Takei, Figwang Fang, Akiko Hirotsu, Moritoki Egi

**Affiliations:** Department of Anesthesia, Kyoto University Hospital, 54 Shogoin- Kawahara-Cho, Sakyo-Ku, Kyoto, 606-8507 Japan

**Keywords:** Neurofibromatosis type 1, Pheochromocytoma, Undiagnosed pheochromocytoma, Spinal surgery, Anesthesia management, Hemodynamic instability

## Abstract

**Background:**

Patients with neurofibromatosis type 1 (NF1) have a 0.1–5.7% incidence of pheochromocytomas. Blood pressure control is difficult in cases of undiagnosed pheochromocytomas without preoperative treatment.

**Case presentation:**

A 39-year-old woman with NF1 and severe cervical deformity presented with progressive quadriplegia and underwent urgent halo-vest placement. A large adrenal mass was incidentally detected on preoperative computed tomography (CT) scans; however, no further examination was performed because the patient had no symptoms suggestive of pheochromocytoma and her cervical spinal condition was considered urgent. When hemodynamic instability occurred during surgery, pheochromocytoma was suspected, and the diagnosis was confirmed postoperatively. Fourteen months later, the tumor was safely resected using strict preoperative management with metyrosine and alpha-blockers and a careful surgical plan.

**Conclusions:**

This case highlights the importance of thorough preoperative screening and the role of anesthesiologists in detecting pheochromocytomas in asymptomatic patients with NF1 who present with intraoperative hemodynamic instability.

## Background

Neurofibromatosis type 1 (NF1) is associated with an increased risk of developing pheochromocytoma [1]. When pheochromocytoma is undiagnosed and untreated before surgery, perioperative blood pressure management becomes particularly challenging and may lead to serious hemodynamic instability.

We report a case in which a giant pheochromocytoma was strongly suspected during spinal fusion surgery in a patient with NF1 based on intraoperative hemodynamic instability and was subsequently confirmed postoperatively.

## Case presentation

**Fig. 1:**
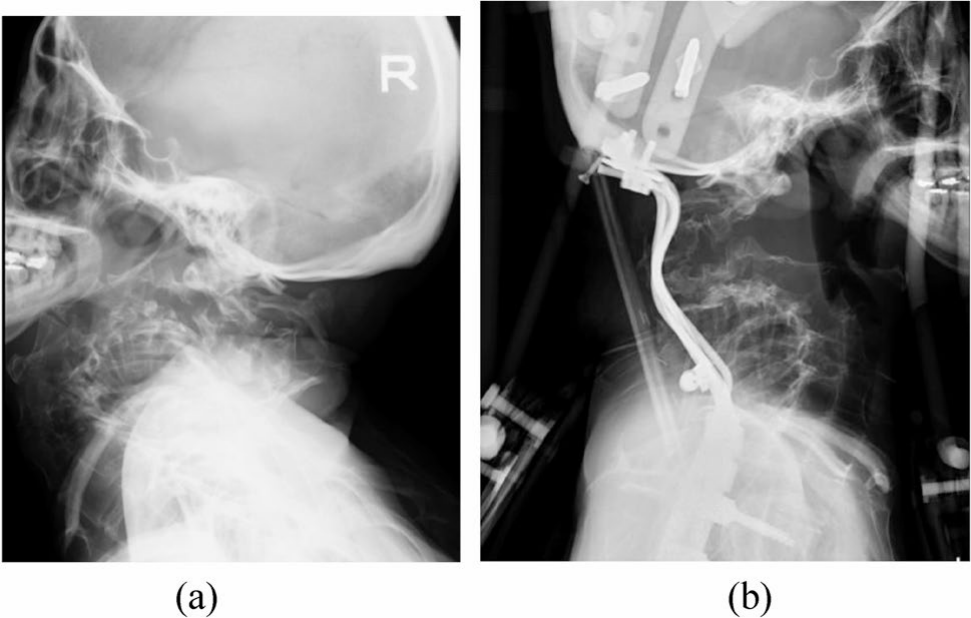
Plain radiographs of the cervical spine. **a** before occipito-thoracic decompression and fusion surgery. **b** after occipito-thoracic decompression and fusion surgery

A 39-year-old woman (height: 138 cm; weight: 38.1 kg) with NF1 and severe cervical kyphosis (Fig. 1) had been followed at a local clinic. Four years earlier, she was referred to orthopedics for bilateral lower limb numbness, and no systemic NF1 screening had been performed. She presented with progressive quadriplegia and underwent urgent halo-vest placement due to spinal cord compression and potential airway compromise. Twenty days later, the patient underwent posterior occipito-thoracic fusion using a right fibular bone graft. Motor-evoked potential (MEP) monitoring was performed during surgery.

The preoperative vital signs were stable (systolic blood pressure (sBP) 100–120 mmHg, heart rate 90–110 bpm), with normal glucose and cardiopulmonary function.

**Fig. 2:**
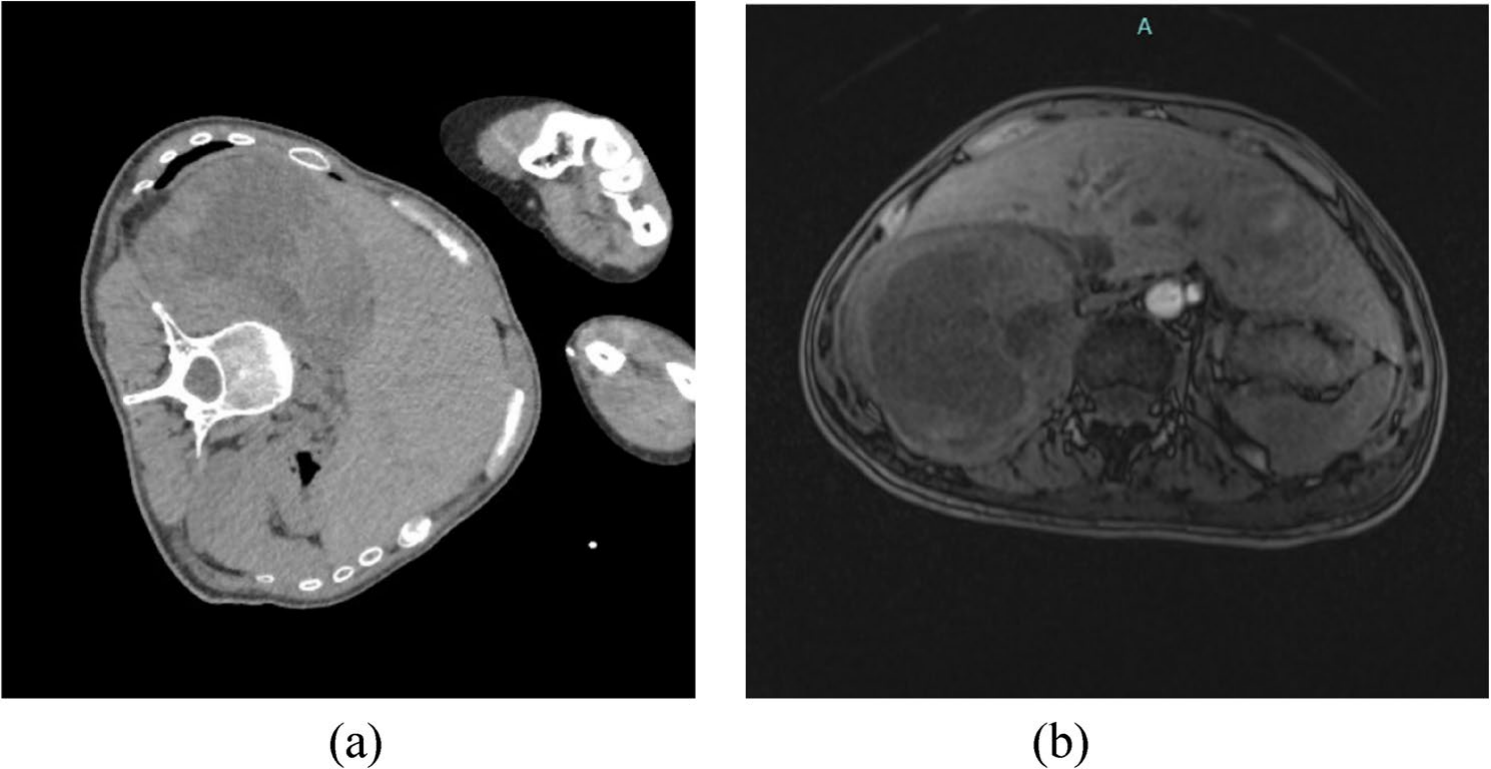
Imaging of the pheochromocytoma. **a** a mass at the upper pole of the right kidney on preoperative CT. **b** a 10 × 10 cm right adrenal mass on MRI

Preoperative chest computed tomography (CT) incidentally revealed a right adrenal mass (Fig. 2a) 20 days before surgery. However, the patient had no symptoms of pheochromocytoma, and surgery was performed without endocrine evaluation because of the urgency of her cervical condition.

Owing to limited neck extension, nasal fiberoptic intubation was performed under sedation with midazolam (2 mg) and fentanyl (50 µg) without a hypertensive response. General anesthesia was maintained using a target-controlled infusion (TCI) of propofol (3–4 µg/mL) and remifentanil (0.09–0.20 µg/kg/min).

After anesthesia induction, blood pressure fluctuations were observed; however, they initially remained responsive to vasopressors and were interpreted as nonspecific hemodynamic changes, such as relative hypovolemia or altered vascular responsiveness. During this early phase, ephedrine and phenylephrine were administered for hypotension.

**Fig. 3:**
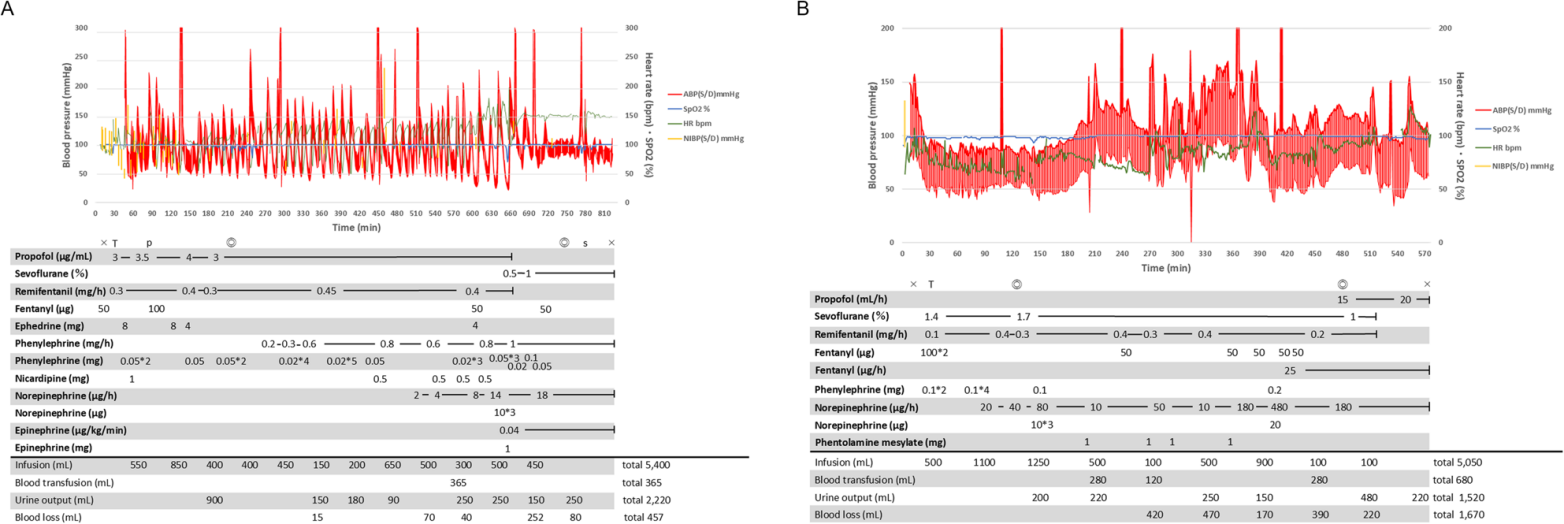
Anesthetic records. **a** Spinal surgery. **b** Pheochromocytoma resection. ×: start of anesthesia and end of anesthesia, ◎: start of surgery and end of surgery. T: tracheal intubation, p: prone position, s: supine position

In retrospect, cyclic fluctuations in blood pressure had already begun before the start of surgery, particularly after prone positioning and during periods of relatively light anesthesia associated with the wake-up test, although these patterns were not clearly recognized at the time. It was not until some time after the start of surgery that we retrospectively recognized, these cyclic and pronounced fluctuations, which persisted and became increasingly difficult to control (Fig. 3a).

Additional vasopressor support with norepinephrine was required; however, despite escalating vasoactive management, hemodynamic instability progressed. Ultimately, the patient’s systolic blood pressure dropped to approximately 30 mmHg, necessitating intravenous epinephrine (1 mg) followed by continuous infusion.

After nearly complete spinal decompression, MEP monitoring was discontinued, and the anesthetic was switched from propofol to sevoflurane. At this stage, the anesthesia team recognized that the cyclic and profound hemodynamic instability was not fully explained by usual anesthetic causes and raised the suspicion of an underlying catecholamine-secreting tumor. The surgical team was immediately consulted, and a shared decision was made to proceed with only the minimum necessary portion of the procedure. This decision was based on balancing the risk of ongoing hemodynamic instability against the risk of neurological deterioration due to incomplete spinal decompression. Given the intraoperative setting, and the need for immediate life-saving management, real-time communication was limited to the surgical team. Therefore, a detailed explanation of the situation was provided to the patient’s family immediately after the surgery.

The patient was transferred to the intensive care unit (ICU), intubated, and sedated, with ongoing circulatory support using continuous infusions of norepinephrine (18 µg/h) and epinephrine (0.04 µg/kg/min). The total duration of anesthesia was 13 h 28 min, with a surgical time of 8 h 58 min. The estimated blood loss was 457 mL, and the urine output was 2,220 mL. Fluid management included 4,650 mL of crystalloids, 750 mL of colloids, and 365 mL of autologous blood via intraoperative cell salvage. Additional fluids and blood products were administered postoperatively to maintain hemodynamic stability.

The patient was extubated on postoperative day one, successfully weaned off catecholamine support, and discharged from the ICU on day two. The halo vest was removed 29 days after surgery with a spinal brace, which was prescribed until bone fusion was confirmed radiographically.

**Fig. 4:**
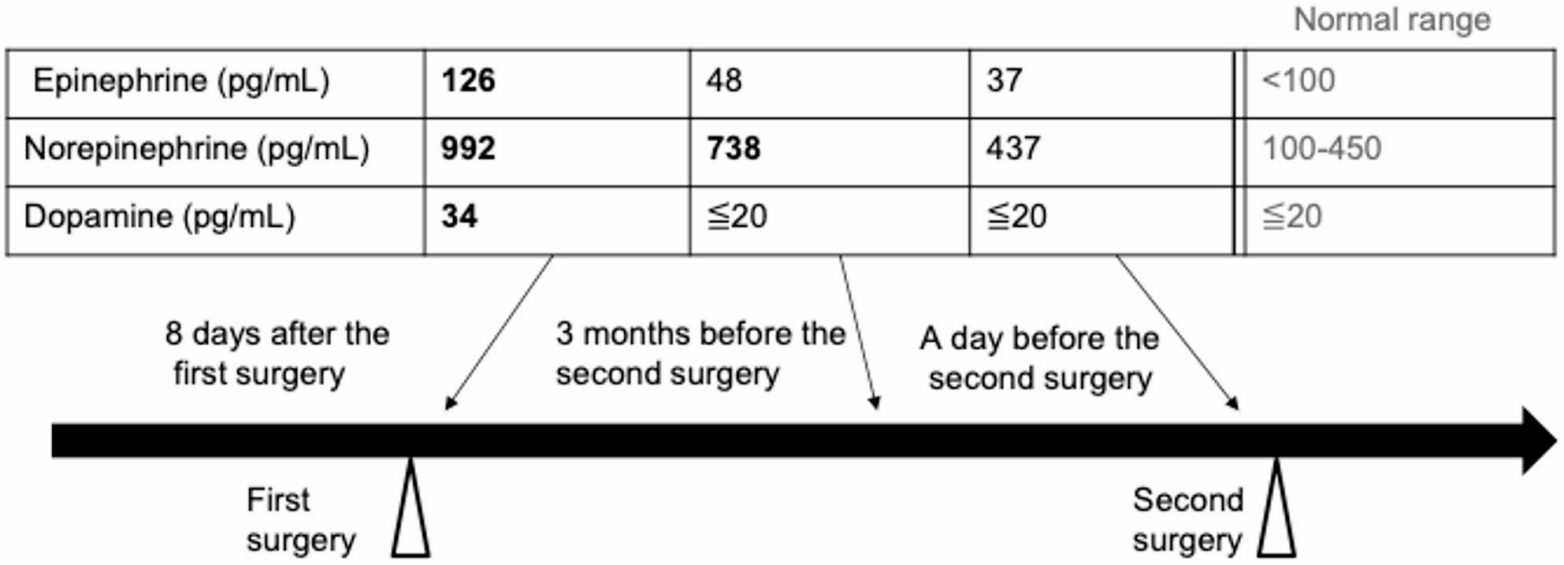
Changes in plasma catecholamine concentrations

The cause of severe intraoperative blood pressure fluctuations was investigated postoperatively. Abdominal magnetic resonance imaging (MRI) revealed a 10 × 10 cm mass in the right adrenal gland (Fig. 2b). Plasma catecholamine concentrations were mildly elevated (Fig. 4), whereas urinary metanephrine (1.1 mg/day; reference range: 0.05–0.2 mg/day) and normetanephrine (5.0 mg/day; reference range: 0.1–0.28 mg/day) were markedly increased, strongly suggesting pheochromocytoma. The patient was discharged on postoperative day 39. After the new-onset palpitations, oral doxazosin mesylate (1 mg/day) was administered. Subsequent I^123^-metaiodobenzylguanidine (MIBG) scintigraphy revealed intense uptake in the right adrenal mass, confirming pheochromocytoma.

Tumor resection was scheduled for 14 months after the initial spinal surgery. Meticulous preoperative preparation included the initiation of metyrosine at 500 mg/day, which was titrated to 2,000 mg/day over 18 days. Doxazosin mesylate was concurrently administered at a dose of 6 mg/day. The day before surgery, the plasma catecholamine levels normalized (Fig. 4). To ensure adequate intravascular volume expansion, 2,000 mL of saline was administered the evening before surgery.

Although the patient reported palpitations preoperatively, no significant fluctuations in blood pressure or glucose levels were noted. General anesthesia was administered for adrenalectomy, and the right adrenal tumor was carefully dissected with minimal direct manipulation to reduce catecholamine surge. Despite these precautions, four episodes of intraoperative hypertension occurred, with the sBP reaching 170 mmHg during tumor handling. Each episode was successfully managed with intravenous phentolamine mesylate (1 mg). Following tumor resection, marked hypotension developed, with sBP dropping to 70 mmHg, requiring norepinephrine infusion (Fig. 3b).

The total surgical time was 6 h 18 min, and the duration of anesthesia was 9 h 34 min. The estimated blood loss was 1,670 mL, requiring a transfusion of 560 mL packed red blood cells and 120 mL fresh frozen plasma. The patient was extubated on postoperative day two, transferred from the ICU to the general ward on day three, and discharged on postoperative day 17.

## Discussion

This case illustrates a rare clinical course in which an asymptomatic pheochromocytoma associated with NF1 was incidentally diagnosed after observing intraoperative cyclic blood pressure fluctuations. This underscores the critical role of anesthesiologists in the perioperative recognition and management of previously undiagnosed pheochromocytomas, particularly in patients with NF1 who may lack the classic symptoms.

NF1 is an autosomal dominant genetic disorder characterized by cutaneous manifestations such as café-au-lait spots and neurofibromas and is associated with an increased risk of various neoplasms, including pheochromocytomas [1, 2].

Pheochromocytomas typically present with headaches, palpitations, and diaphoresis; however, they may remain asymptomatic, as in this case. Asymptomatic tumors are often discovered incidentally through imaging, rendering symptom-based diagnoses unreliable [3]. In this patient, although a right adrenal mass was visible on preoperative CT, it was not fully evaluated because of the absence of symptoms and the urgency of her cervical spinal condition.

While a review study in patients with NF1 reported a pheochromocytoma prevalence of 0.1–5.7% [1], a prospective study found a prevalence of 7.7%, with more than half of cases being asymptomatic, underscoring the limitations of relying solely on clinical symptoms [4]. Similarly, a case report described a patient with NF1 whose adrenal tumor was incidentally detected during hospitalization for abdominal pain and was later confirmed as a pheochromocytoma on imaging performed during routine evaluation [5]. Another report [6] described a patient who underwent nonadrenal surgery and experienced severe intraoperative hypertension, leading to a postoperative diagnosis of occult pheochromocytoma. In both the previous report and the present case, the absence of preoperative symptoms and lack of biochemical screening resulted in an unrecognized pheochromocytoma prior to surgery. Collectively, these findings highlight the importance of routine screening for pheochromocytoma in NF1 patients, even in those without typical symptoms.

In the present case, the diagnosis was prompted by cyclic blood pressure fluctuations after anesthesia induction, which could not be fully explained by vasoactive agents alone and suggested intermittent catecholamine release. Similar patterns have been reported in pheochromocytoma, including during nonadrenal surgery, and may be triggered by hemodynamic changes, vasopressor use, or mechanical stimulation such as prone positioning [7, 8]. The hemodynamic instability in this case was likely multifactorial. Chronic catecholamine excess may have reduced effective circulating volume, which was further exacerbated by preoperative fasting and anesthetic-induced vasodilation. In addition, vasopressor administration, prone positioning, and relative light anesthesia during the wake-up test may have contributed to unstable catecholamine release and cyclic blood pressure fluctuations.

A key issue was not only recognizing this abnormal pattern but also determining how to proceed under suspected but unconfirmed pheochromocytoma. Although an adrenal mass was visible on preoperative CT, it had not been evaluated further due to the absence of symptoms and the urgency of the spinal condition. Furthermore, the anesthesiologists’ attention was intensely focused on the difficult airway management required for awake intubation due to the patient wearing a halo vest, which contributed to the oversight of the adrenal mass on the preoperative CT. The anesthesiologist initially interpreted the instability as nonspecific; however, after the development of cyclic and pronounced fluctuations following prone positioning, a catecholamine-secreting tumor was suspected.

Although postponement is generally recommended in such situations [9], this surgery was considered semi-emergent due to progressive neurological deficits. Weighing the life-threatening hemodynamic instability against the high risk of permanent neurological deterioration, and after discussion with the surgical team, a decision was made to complete only the minimum necessary decompression and terminate the procedure early. Retrospectively, earlier recognition and re-evaluation of preoperative imaging might have led to postponement. In retrospect, when significant hemodynamic instability occurs after anesthesia induction, the operation should be paused rather than continued without adequate preoperative preparation. In patients with pheochromocytoma, failure to recognize and manage catecholamine-secreting tumors before surgical stimulation may induce a severe hypertensive crisis, increasing the rate of perioperative complications and mortality [9].

Therefore, although pheochromocytoma has not been confirmed, surgery in such unstable conditions is hazardous and deviates from the recommended perioperative management. Intraoperative management required substantial fluid resuscitation (4,650 mL of crystalloid and 750 mL of colloid) to compensate for the chronic hypovolemia associated with untreated pheochromocytoma. This volume deficit may have contributed to the hemodynamic instability. Adequate preoperative preparation, including alpha-adrenergic blockade and catecholamine synthesis inhibition, is essential for reducing perioperative cardiovascular complications in patients with pheochromocytoma [10, 11]. In this case, preoperative metyrosine was carefully titrated, and intraoperative hemodynamics were controlled with phentolamine for hypertension and norepinephrine for hypotension. Despite the tumor’s proximity to vital structures, surgical resection was safely performed under well-controlled circulatory conditions, highlighting the effectiveness of coordinated, multidisciplinary perioperative planning.

Similar cases have been reported [7, 8], but this case emphasizes the risk of a missed diagnosis in asymptomatic NF1 patients. It also highlights the role of anesthesiologists in recognizing characteristic hemodynamic patterns and the importance of integrating physiological findings, imaging results, and surgical urgency into intraoperative decision-making.

In conclusion, this case underscores the importance of thorough preoperative screening and careful imaging review, even in asymptomatic NF1 patients, as pheochromocytomas may remain clinically silent and lead to delayed or missed diagnoses. This highlights the critical role of anesthesiologists in identifying the underlying pathology of unexplained intraoperative hemodynamic instability. Furthermore, the successful perioperative use of combined α-adrenergic blockade and metyrosine demonstrated the effectiveness of this approach in managing large pheochromocytomas and ensuring safe surgical outcomes. 

## Data Availability

The relevant data for this case report are unavailable for public access because of patient privacy concerns.

## Abbreviations

NF1: Neurofibromatosis type 1
CT: Computed tomography
MRI: Magnetic resonance imaging
ICU: Intensive care unit
sBP: Systolic blood pressure
bpm: Beats per minute
MEP: Motor evoked potential
TCI: Target-controlled infusion
MIBG: Metaiodobenzylguanidine
